# The effect of feedback timing on category learning and feedback processing in younger and older adults

**DOI:** 10.3389/fnagi.2024.1404128

**Published:** 2024-06-03

**Authors:** Kristen Nunn, Robert Creighton, Victoria Tilton-Bolowsky, Yael Arbel, Sofia Vallila-Rohter

**Affiliations:** ^1^MGH Institute of Health Professions, Boston, MA, United States; ^2^Geriatric Research Education and Clinical Center, VA Pittsburgh Healthcare System, Pittsburgh, PA, United States; ^3^Johns Hopkins University School of Medicine, Baltimore, MD, United States

**Keywords:** feedback, timing, event-related potentials, aging, category learning

## Abstract

**Introduction:**

Corrective feedback can be received immediately after an action or with a temporal delay. Neuroimaging studies suggest that immediate and delayed feedback are processed by the striatum and medial temporal lobes (MTL), respectively. Age-related changes in the striatum and MTL may influence the efficiency of feedback-based learning in older adults. The current study leverages event-related potentials (ERPs) to evaluate age-related differences in immediate and delayed feedback processing and consequences for learning. The feedback-related negativity (FRN) captures activity in the frontostriatal circuit while the N170 is hypothesized to reflect MTL activation.

**Methods:**

18 younger (*M_years_* = 24.4) and 20 older (*M_years_* = 65.5) adults completed learning tasks with immediate and delayed feedback. For each group, learning outcomes and ERP magnitudes were evaluated across timing conditions.

**Results:**

Younger adults learned better than older adults in the immediate timing condition. This performance difference was associated with a typical FRN signature in younger but not older adults. For older adults, impaired processing of immediate feedback in the striatum may have negatively impacted learning. Conversely, learning was comparable across groups when feedback was delayed. For both groups, delayed feedback was associated with a larger magnitude N170 relative to immediate feedback, suggesting greater MTL activation.

**Discussion and conclusion:**

Delaying feedback may increase MTL involvement and, for older adults, improve category learning. Age-related neural changes may differentially affect MTL- and striatal-dependent learning. Future research can evaluate the locus of age-related learning differences and how feedback can be manipulated to optimize learning across the lifespan.

## Introduction

Learning occurs throughout the lifespan and is often an error-ridden process. As new learners make errors, error-detection plays a key role in updating incorrect associations in memory (Luft, 2014). Error detection is the recognition that an action conflicts with what is true relative to internal or external criteria (Ohlsson, 1996; Postma, 2000). Detection of errant behavior can be either internally (i.e., self-monitoring) or externally (i.e., feedback) driven. External error detection via feedback is critical when learners are acquiring new information or are unable to monitor the accuracy of their own responses (McCandliss et al., 2002; Pashler et al., 2005). For example, when learners are acquiring new phonological contrasts (e.g., Japanese speakers learning an English /r/ - /l/ distinction; McCandliss et al., 2002) or in certain cases of cognitive and/or linguistic deficit such as aphasia, Alzheimer’s, and aging (Schreiber et al., 2011; Nitta et al., 2017; Mandal et al., 2020). Thus often, feedback is not only helpful, but critical to the process of learning.

Learning conditions can influence how feedback is detected, processed, and utilized to update memory. Feedback timing (immediate vs. delayed) is one such condition of relevance to the current work. Immediate feedback is hypothesized to recruit dopamine-dependent striatal circuits which code prediction errors and send reward signals to the anterior cingulate cortex (ACC) (Holroyd and Coles, 2002; Nieuwenhuis et al., 2004). When feedback provision is delayed, (≥ 3,500 ms) the fast-acting dopamine-mediated learning is disrupted and processing shifts to the medial temporal lobe (MTL) which supports binding information that is separated by time (Foerde and Shohamy, 2011; Peterburs et al., 2016; Arbel et al., 2017).

Event-related potentials (ERPs) collected using electroencephalography (EEG) have been leveraged to elucidate the differences in the processing of feedback during learning. The feedback-related negativity (FRN) is a frontocentral negativity that peaks 250-300 ms after the provision of feedback (Gehring et al., 1995; Miltner et al., 1997).^1^ The FRN is hypothesized to capture immediate feedback processing within the fronto-striatal circuit (Holroyd and Coles, 2002; Nieuwenhuis et al., 2004). The amplitude of the FRN is sensitive to feedback valence (negative > positive) (Gehring et al., 1995; Miltner et al., 1997) and feedback timing (immediate > delayed). Reductions in the FRN amplitude in response to delayed feedback is consistent with the hypothesis that delays in feedback timing shift processing away from fronto-striatal circuits (Weinberg et al., 2012; Peterburs et al., 2016; Weismüller and Bellebaum, 2016; Arbel et al., 2017; Kim and Arbel, 2019).

The N170 (Bentin et al., 1996) has been used to evaluate the processing of delayed feedback. The N170 is larger for delayed relative to immediate feedback (Arbel et al., 2017; Kim and Arbel, 2019; Höltje and Mecklinger, 2020; Albrecht et al., 2023) and in the context of feedback-based tasks has been hypothesized to reflect activity in the medial temporal lobe (MTL) (Arbel et al., 2017; Kim and Arbel, 2019; Höltje and Mecklinger, 2018, 2020; Albrecht et al., 2023). This is supported by neuroimaging studies that find that the MTL activation is heighted by delayed feedback (Foerde and Shohamy, 2011; Lighthall et al., 2018) and double dissociations in which individuals with MTL damage have been found to learn from immediate but not delayed feedback with the opposite pattern observed in individuals with basal ganglia damage due to Parkinson’s Disease (Foerde et al., 2013). Outside of the context of feedback-based learning, the N170 has been hypothesized to reflect activity in the MTL (Grippo et al., 1996; Baker and Holroyd, 2013) as well as the adjacent fusiform gyrus (Iidaka et al., 2006; Rossion and Jacques, 2011; Gao et al., 2019). Grippo et al. (1996) associated the N170 with the MTL when they observed a reduction in the amplitude of the N170 with increasing memory load in patients with temporal lobe epilepsy. More recently, Baker and Holroyd (2013) used source localization algorithms to localize the N170 to the MTL during a spatial navigation task. Similar activation during the processing of complex objects is localized to the fusiform gyrus (Iidaka et al., 2006; Rossion and Jacques, 2011). The N170, however, is not restricted to the visual domain and has been found to be elicited and modulated by the timing of auditory feedback; further supporting the notion that in the context of feedback-based learning, the N170 reflects cognitive processes that are not specific to the visual domain (Kim and Arbel, 2019). Albrecht et al. (2023) suggests that it is possible there are two potentially overlapping components that reflect activity in the MTL and fusiform gyrus, respectively. Our decision to use the N170 to gain insight into processes that are potentially supported by the MTL is motivated by (1) research finding that delayed feedback is associated with greater MTL activation, (2) previous studies identifying the MTL as a generator of the N170, and (3) research indicating that the N170 can be elicited by auditory feedback, and thus, likely does not reflect visual processing in the fusiform gyrus when elicited by feedback. In the current study, a larger amplitude N170 in the delayed relative to immediate feedback condition will support the claim that the N170 may reflect processing in the MTL although this cannot be definitively stated in the absence of source localization data which is beyond the scope of the current study.

Aging is associated with changes in striatal and MTL functioning and may influence older adults’ ability to learn successfully from certain types of feedback. Characterizing how age-related changes in neural functioning influences learning in older adults is key to supporting learning across the lifespan and may be particularly useful in rehabilitation contexts. Rehabilitation services, such as speech-language therapy, are primarily provided to adults over the age of 60 (American Speech-Language Hearing Association, 2019), many of whom have experienced an acquired brain injury (e.g., stroke, traumatic brain injury, tumor resection). Research in rehabilitation continually aims to improve the effectiveness of interventions and retention of treatment gains which may be informed by understanding how age-related changes in neural functioning interact dynamically with acquired neurologic damage.

In older adults, memory decline has been found to be greater in declarative memory tasks that require recruitment of the MTL relative to non-declarative tasks that require the recruitment of the striatal circuit (Hoyer and Verhaeghen, 2006). This is consistent with findings of a more accelerated loss of volume in the MTL relative to the striatum in later life (Raz et al., 2005, 2010; Walhovd et al., 2011). Yet, age-related changes in the striatum are also identified and have been evaluated in the context of reward processing (Mell et al., 2005; Bäckman et al., 2006; Eppinger et al., 2008, 2013; Braver, 2012; Chowdhury et al., 2013; Samanez-Larkin et al., 2014). Older adults show reduced neural response to reward signals such as feedback and differences in the processing of negative and positive rewards relative to younger adults (Holroyd and Coles, 2002; Nieuwenhuis et al., 2002; Eppinger et al., 2008, 2013). Importantly, age-related differences in reward processing have been found to influence learning (Eppinger et al., 2013). Evaluating learning in older adults from immediate and delayed feedback may elucidate how age-related changes in the striatum and MTL effect feedback-based learning.

Only one study (Lighthall et al., 2018) to our knowledge, has evaluated learning from immediate and delayed feedback in older adults to determine whether this manipulation alters learning outcomes and neural activity. Lighthall et al. (2018) evaluated probabilistic learning and recognition memory in immediate (1,000 ms after response) and delayed (7,000 ms after response) feedback conditions. They compared younger (*M_years_* = 26.3) and older adults (*M_years_* = 68.7) to better understand the effects of healthy aging on feedback processing. Behavioral accuracy analyses demonstrated that older adults showed lower rates of optimal response selection relative to young adults, but that both groups showed learning under both timing conditions. Region of interest analyses of functional magnetic resonance imaging data (fMRI) collected during learning demonstrated greater activity in the striatum relative to the hippocampus with immediate feedback in the young adult group, and the opposite pattern with delayed feedback. In contrast, in the older adult group, feedback timing did not lead to significant differences in regional activation during learning. Additional analyses focused on the nucleus accumbens, a region important for dopaminergic reward learning, identified enhanced activation for both groups under conditions of immediate feedback relative to delayed feedback. The authors concluded that findings provide evidence for age-related change in hippocampal mechanisms of learning more so than in striatal mechanisms.

The current study will characterize age-related differences in category learning with immediate and delayed feedback. During learning, individual’s electrophysiological response to feedback will be captured using ERPs. ERPs provide high-temporal resolution of the processing of feedback under different timing conditions. Category learning is central to human cognition and broadly defined as the ability to organize environmental information into meaningful groups based on patterns (Ashby and O’Brien, 2005). Abstract representations of a concept or “prototypes” can aid in categorization. In A/B prototype category learning, category exemplars are derived from an “A” prototype and “B” prototype. “A” category members share relatively more features with prototype “A” while “B” category members share relatively more features with prototype “B.” For example, an email may be categorized as phishing because it shares relatively more features with a scam email (from a financial institution, implies urgency, requests personal information, contains typos) relative to an official business email. To our knowledge, no studies have evaluated Prototype A/B category learning under immediate and delayed feedback. The ubiquity of category learning and the novelty of this investigation further supports the impact of the current work.

Consistent with previous research (Lighthall et al., 2018) we predict that learning outcomes will be equivalent under both immediate and delayed feedback timing conditions but be associated with different signatures of neural activity. In younger adults, we expect to see a larger FRN in response to immediate feedback relative to delayed feedback and a larger N170 in response to delayed relative to immediate feedback. As is characteristic of the FRN, we expect a larger magnitude FRN to negative relative to positive feedback (Sambrook and Goslin, 2015) in both the immediate and delayed conditions. Yet, we recognize that a disruption of feedback processing in the striatum may also be evidenced by the absence of a valence effect in the delayed condition (valence by timing interaction) indicating atypical extraction of outcome-related information conveyed by feedback. We expect to see the same pattern in the FRN for older adults. However, a larger magnitude N170 in response to delayed relative to immediate feedback may be absent in older adults with this affect potentially related to age-related changes in the MTL. We do not expect to see a valence effect in the N170 because the N170 is hypothesized to reflect the binding of temporally discontiguous events and not the extraction of outcome information conveyed via feedback. The findings of this study aim to further characterize consequences of age-related neural changes on feedback-based learning, setting foundations for future research examining how age-related changes in neural functioning may interact with learning after acquired neurologic injury.

## Methods

### Participants

Thirty-eight adults, 18 younger (Female = 13, Male = 5) and 20 older (Female = 15, Male = 5) participated in the study. A power analysis based on previous research evaluating the effect of manipulating feedback timing on the magnitude of the N170 and the FRN (Arbel et al., 2017; Kim and Arbel, 2019; Höltje and Mecklinger, 2020) indicated that the current sample size would enable the detection of the main effect of feedback timing on ERP amplitude with power > 0.95 and α = 0.05. Younger adults ranged from 22–30 years old (*M* = 24.4, *SD* = 2.5). Older adults ranged from 55 to 82 years old (*M* = 65.5, *SD* = 6.3). 55 years old was selected as the lower bound for older adults given changes in neural function and structure that accelerate around 50-years-old (Dohm-Hansen et al., 2024) including volume loss in hippocampal and adjacent parahippcampal regions (Fjell et al., 2013; Luo et al., 2020). The mean age of this sample is comparable to other studies evaluating feedback processing in younger and older adults (Nieuwenhuis et al., 2002; Eppinger et al., 2008; Lighthall et al., 2018). Participants did not have a history of developmental delay, neurologic impairment, or learning disability. All participants scored in the ‘no cognitive impairment’ range of the Mini-Mental Status Examination (MMSE, Folstein et al., 1975). Three older adults and one younger adult were excluded from the EEG analysis due to technical errors or excessive artifacts. Thus, 17 younger (*M* = 24.2, *SD* = 2.4) and 17 older adults (M = 64.4, *SD* = 4.7) were included in the EEG analysis.

### Procedure

The study procedure was approved by the Institution Review Board of Mass General Brigham Healthcare System. All procedures were completed in a quiet room at the MGH Institute of Health Professions in one 2–3-h session.

#### Category learning task

**Figure 1:**
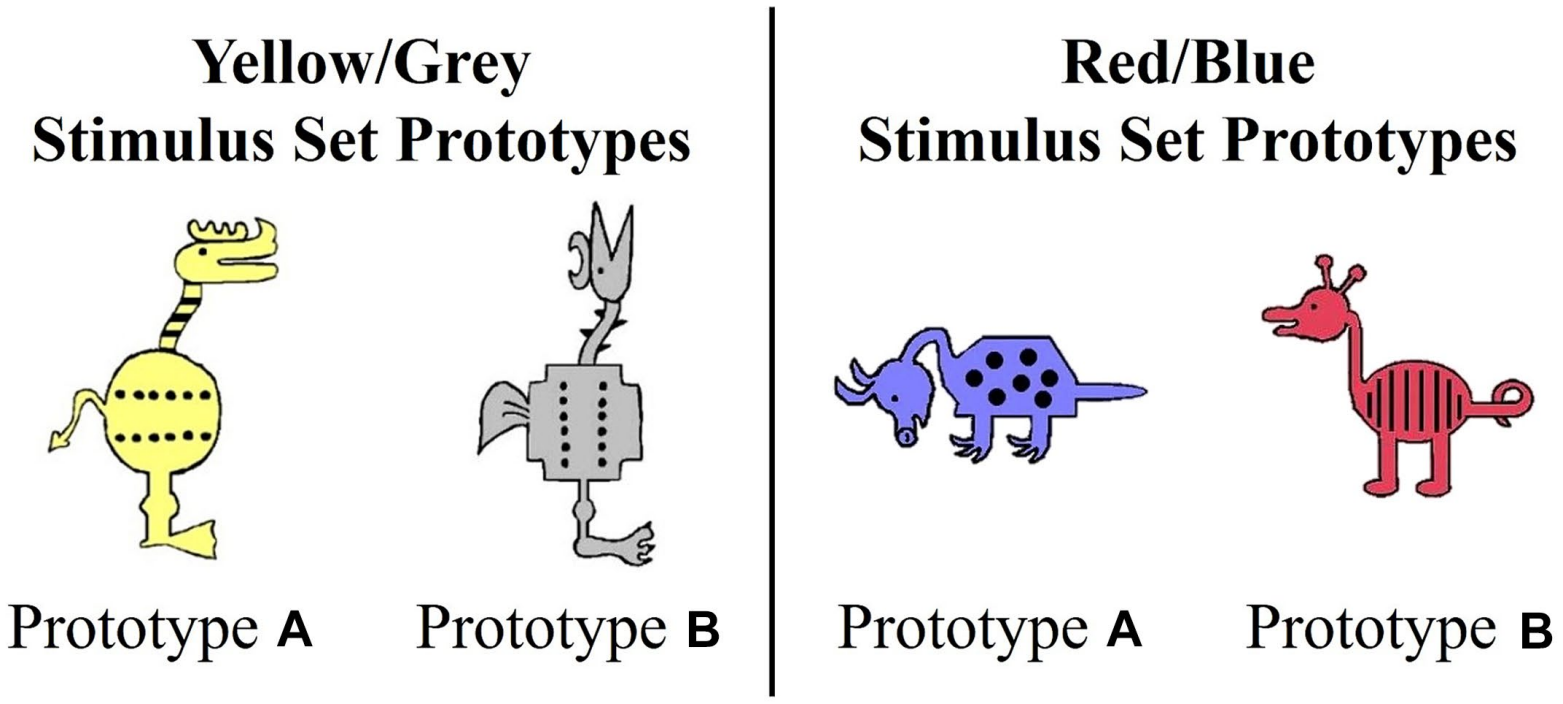
Prototypes for yellow/gray and red/blue stimulus sets.

Participants completed two Prototype A/B learning tasks administered using E-Prime (Psychology Software Tools Inc, 2016) across two separate learning blocks, one with immediate and one with delayed feedback separated with a break. We used two stimulus sets, a yellow/grey set and red/blue set, that were developed by Reed et al. (1999) and adapted by Zeithamova et al. (2008) (Figure 1). We crossed stimulus sets with timing conditions to create four tasks: red/blue immediate, red/blue delayed, yellow/grey immediate, and yellow/grey delayed. Each participant completed one training block with immediate feedback followed by testing and one training block with delayed feedback followed by testing. During each training block, participants saw a different stimulus set. The order of the blocks was counterbalanced across participants.

**Figure 2:**
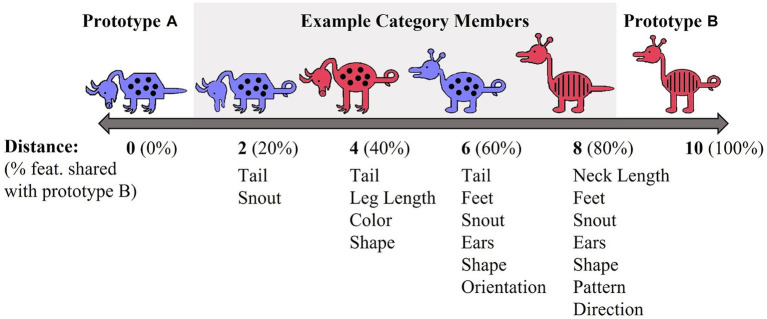
Prototype A/B learning task category structure.

Each stimulus set varied on 10 binary dimensions (Zeithamova et al., 2008; Vallila-Rohter and Kiran, 2013). For example, for the red/blue stimulus set, binary dimensions included: animal’s neck (short vs. long), tail (straight vs. curly), feet (pointed vs. curved), snout (pointed vs. rounded), ears (pointed vs. rounded), color (blue vs. red), body shape (pyramidal vs. round), body pattern (spots vs. stripes), head orientation (downward-facing vs. upward-facing), and leg length (short vs. long). Prototypes A and B were maximally distinctive and differed on all 10 binary features. The stimulus dimensions of the yellow/grey stimulus set were visually distinct from the red/blue set. Category membership was determined by an animal’s distance from the prototype (Figure 2). Animals that shared 90–60% of their features with a prototype were considered members of that prototype category. Animals that differed by five features with both prototypes were considered ambiguous and could be correctly categorized into either category. The category structure creates a continuum in which exemplars share 10–40% of their similarity with the opposing prototype. As is typical with Prototype learning tasks, the number of dimensions (*n* = 10) upon which category members vary likely exceeds working memory capacity and thus, optimal categorization requires that individuals use feedback to acquire cue-outcome relationships among the stimulus dimensions that cannot be easily verbalized (Ashby and Ell, 2001).

#### Training

**Figure 3:**
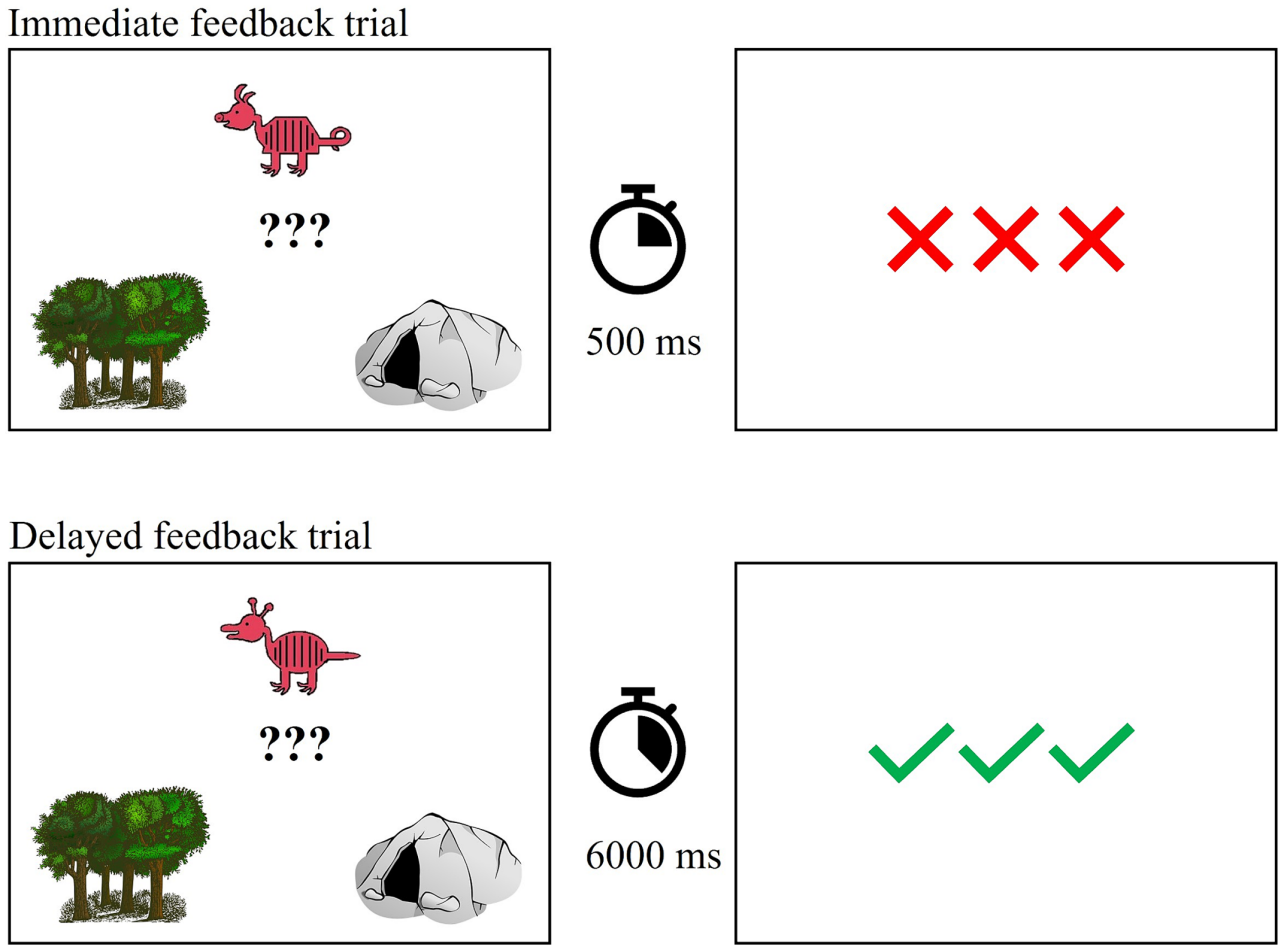
Example training trial with immediate and delayed feedback.

Prior to training, individuals were instructed to base their decisions on the overall appearance of each animal rather than on one or two features. Training consisted of 80 trials in each task which is sufficient for learning (see Zeithamova et al., 2008; Vallila-Rohter and Kiran, 2013; Tilton-Bolowsky et al., 2021). Of note, four participants completed 60 training trials due to a coding error. The number of unique animals presented during training and their distance from the prototype were consistent with Zeithamova et al. (2008). Participants saw 10 unique animals from category A (2 each at distances of 1 and 4, 3 each at distances of 2 and 3) and 10 unique animals from category B. Each exemplar was presented four times. Prototypical animals never appeared in training. Participants were asked to decide whether each animal lived in the forest (i.e., category A) or in a cave (i.e., category B). In each trial, participants saw one exemplar and a drawing of a forest and a cave. After making a response via button press, participants received feedback immediately (500 ms) or after a delay (6,000 ms). Feedback was in the form of three green checks (correct) and three red X’s (incorrect). If participants did not respond within 4,000 ms, they saw a drawing of an hourglass and were instructed to respond faster. Figure 3 shows an example training trial.

#### Testing

Testing consisted of 28 trials. The trial structure of testing was identical to training except participants did not receive feedback on their response accuracy. During testing, participants saw 13 exemplars in category A (1 prototype and 3 each at distances of 1, 2, 3 and 4), 13 exemplars in category B, and 2 ambiguous exemplars (distance of 5). A total of 6 stimuli had been included in training while 22 were untrained. The testing list was designed so that participants saw each binary feature (e.g., red/blue) an equal number of times.

### Data analysis

#### Behavioral data

Learning performance was evaluated using slope scores. Slope scores were calculated using a percent “B” response (%BResp) (see Vallila-Rohter and Kiran, 2013). %BResp shows participant accuracy as a function of distance from the prototype and accounts for learners’ tendency to “probability match” or respond in proportion to the probability that each stimulus–response feature is reinforced during learning (Knowlton et al., 1994; Vallila-Rohter and Kiran, 2013). Similar to prior studies, the %BResp was predicted to increase 10% for each feature shared between the exemplar and prototype B (Vallila-Rohter and Kiran, 2013). For example, an animal that differs by one feature from the prototype A would have a predicted 10%BResp because it shares 10% of its features with prototype B. Within this model, successful category learning would correspond to a %BResp with a linearly increasing slope of 10. Chance response corresponds to a slope of zero in which participants have a 50%BResp at each distance from the prototype. Prior work has identified that when participants make responses based on multiple features, slope scores approach 10 more so than strategies where one feature determines categorization or a random approach is utilized (Vallila-Rohter and Kiran, 2015).

To determine whether individual results were linear, significance levels of regressions for each participant were compared when the independent variable of distance was squared, cubed, and unadjusted. When the non-squared regression reached significance with an alpha value <0.05 and the significance of the squared and cubed terms exceed 0.05, the data was considered linear (Cox and Wermuth, 1994; Gasdal, 2012). Regression lines were fitted to participant results and regression coefficients were used as slope scores. The resultant slope scores reduced the linearly increasing %BResp scores at each distance into a single score where 10 represented optimum performance.

#### EEG data processing and analysis

A 32-channel GES 400 System by Electrical Geodesics Inc. was used to obtain EEG data with a 32-channel HydroCel Geodesic sensor net. The net comprised of Ag/AgCl electrodes attached to an elastic net consistent with the international 10–20 system. Per manufacture recommendations, impedances were kept below 50 kΩ. Offline analysis of the EEG data included bandpass filtering (0.1-30 Hz) of raw data and segmentation into 1,000 ms long epochs (200 ms before and 800 ms after feedback presentation). Body movement artifacts were rejected by visually inspecting each trial for drift. Re-referencing to the average reference was performed followed by baseline correction using the signal 200 ms prior to the presentation of feedback. Independent component analysis (ICA) was used to remove ocular, muscular, and other artifacts.

EEG was recorded during training. Consistent with previous research, the FRN was captured at fronto-central electrode, FCz and the N170 at occipital-parietal electrodes, P7 and P8. To allow for the analytic reduction of the temporality of the data (Spencer et al., 2001), averaged data from each participant was submitted to a Temporal Principal Component Analysis (TPCA) using EEGLAB (Delorme and Makeig, 2004). TPCA was used to overcome challenges with other ERP analysis methods (e.g., difference waves and mean amplitude) (Dien, 2012). TPCA eliminates the need to select a time window for analysis or calculate difference waves, two aspects of ERP analysis that can bias results (Luck and Gaspelin, 2017) and are relevant to the current study. TPCA decomposes the observed signal into underlying factors representing comparable activity patterns across trials thus eliminating the need to specify a time window for analysis. This is particularly useful for the current study, given that the peak amplitude latency can vary across younger and older adults (e.g., Eppinger et al., 2008; Yi et al., 2012). TPCA also evades the need to take difference waves which requires the subtraction of two conditions (e.g., positive and negative feedback). If the two subtracted waves differ by more than just magnitude but also the kind of signal conveyed or peak latency, the resultant difference wave can produce misleading results (Dien and Frishkoff, 2005)TPCA allows for the disentanglement of underlying components that overlap in the time domain and is well-fit for the current study (Dien, 2012; Scharf et al., 2022). TPCA was conducted for each electrode, FCz, P7, and P8. We identified the temporal factor with peak latencies which overlapped with the FRN and N170 grand average waveforms. The corresponding factor scores were extracted. Factor scores reflected the relative level of magnitude of the FRN and N170 for each participant at a given electrode.

#### Statistical analysis

All statistical analyses were performed in R (R Core Team, 2021). To evaluate whether learning slope scores varied across groups and feedback-timing conditions, a 2 (group: younger adult vs. older adult) by 2 (feedback timing: immediate vs. delayed) mixed analysis of variance (ANOVA) with slope scores as the dependent variable was conducted. To evaluate the relationship between group, feedback timing, feedback valence, and ERP magnitude, we planned to conduct three 2 (group: younger adult vs. older adult) by 2 (feedback timing: immediate vs. delayed) by 2 (feedback valence: positive vs. negative) mixed ANOVAs with factor scores derived from TPCA as the dependent variables. Because a separate TPCA was performed for each electrode, the resultant factor scores cannot be compared across electrodes and thus, were analyzed in separate ANOVAs.

## Results

### Behavioral data

During testing, participants’ mean response accuracy (number of exemplars categorized correctly/total number of trials) was above chance for the immediate (younger adults: *M* = 71.4, *SD* = 9.3; older adults: *M* = 60.9, *SD* = 18.1) and delayed conditions (younger adults: *M* = 70.4, *SD* = 9.7 older adults: *M* = 68.9, *SD* = 12.3). Reaction time was numerically shorter in the immediate (younger adults: *M* = 765.9, *SD* = 341.3; older adults: *M* = 976.6, *SD* = 345.0) compared to the delayed condition (younger adults: *M* = 907.5, *SD* = 405.1; older adults: *M* = 1009.5, *SD* = 331.6). During testing participants, on average, did not respond to <1 trial in the immediate (younger adults: *M* = 0.39; older adults: *M* = 0.40) and delayed conditions (younger adults: *M* = 0.33; older adults: *M* = 0.30). This rate was comparable across timing and age groups.

**Figure 4:**
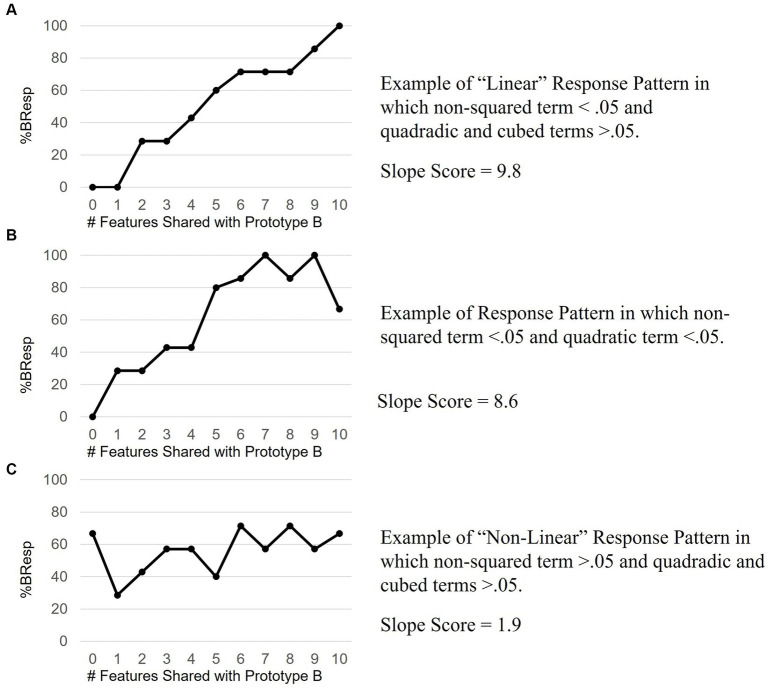
Example response patterns. **Panel** **A** shows an ideal linear response pattern reflective of learning. **Panel** **B** data align with both linear and quadratic trends suggesting an incomplete increase in %B response with increasing distance from Prototype B. **Panel** **C** data do not align with linearly increasing, quadratic or cubed trends suggestive of a random response pattern, or no learning.

Pairwise comparisons with Holm correction revealed a steadily increasing %BResp with each increase in distance from Prototype A. This is consistent with the prediction that participants will probability-match their responses based on how many features an exemplar shares with the prototype and supports the decision to analyze %BResp data over accuracy data which does not reflect this prediction. 50 out of 76 task response patterns met the criteria used to assess the assumption of linearity confirming that the predominant relationship between distance and %BResp was linear. 7 of the 26 response patterns had significant non-squared and quadratic and/or cubic terms and thus, in our conservative classification, were not considered “linear” even though these response patterns demonstrated an increase in %BResp when an exemplar shared more features with Prototype B. 57 out of 76 (75%) of participants showed evidence of an increasing %BResp when an exemplar shared more features with Prototype B. See Figure 4 for examples of response patterns that met the criteria for “linear” and “non-linear.” Figure 4B shows an example of a response pattern in which the non-squared term was significant as well as the quadratic term.

**Figure 5:**
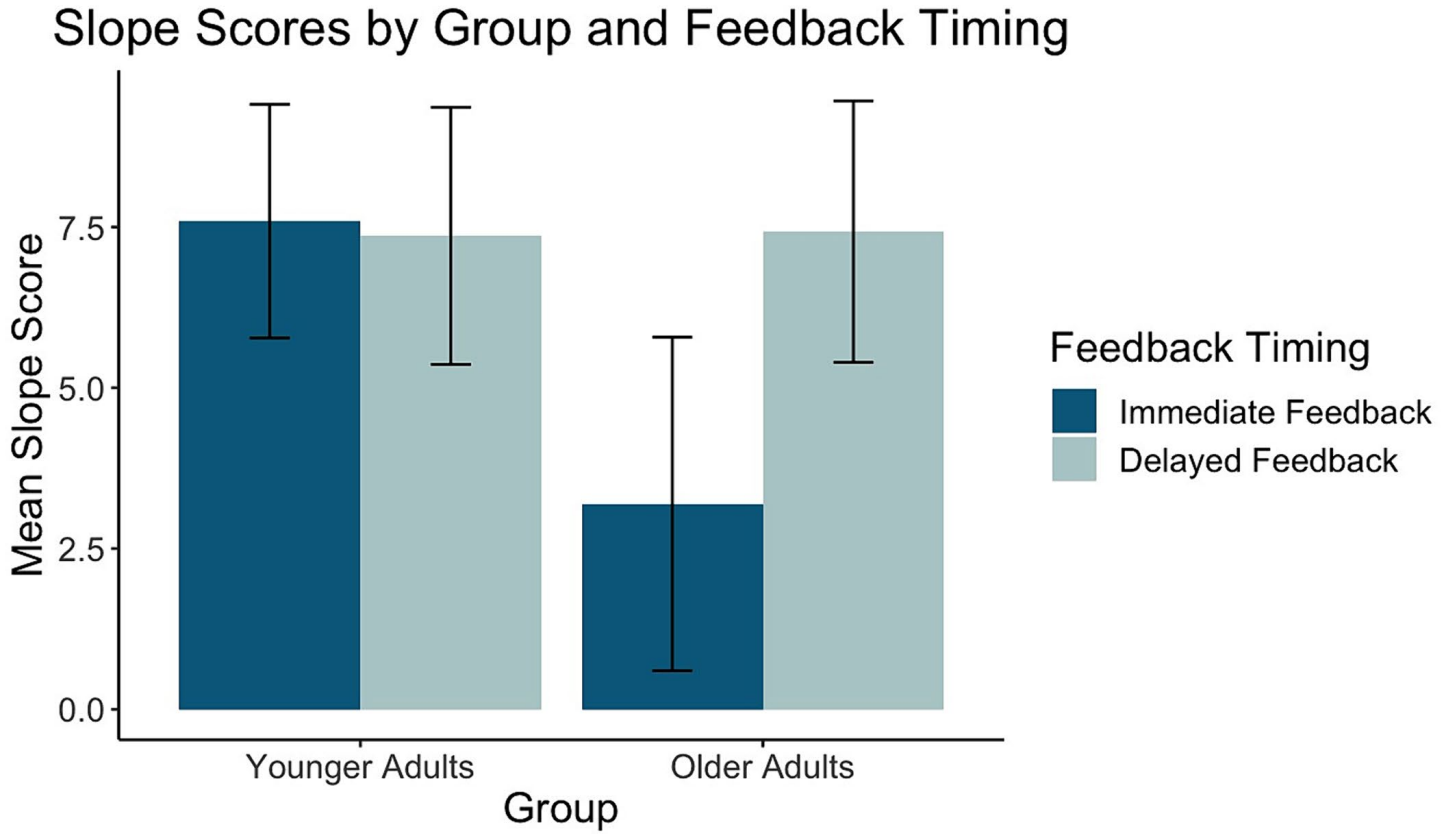
Mean slope scores for younger and older adults across feedback timing conditions.

Of the 26 task response patterns that did not meet the criteria used to assess the assumption of linearity 14 were in the immediate condition (5 younger adults, 9 older adults) and 12 in the delayed condition (5 younger adults, 7 older adults). Five individuals (3 younger adults, 2 older adults) had response patterns failing to meet the criteria for the assumption of linearity across both the immediate and delayed tasks. Our evaluation of the data revealed that the predominant pattern was linear and that slope scores reflected the degree to which individuals learned the overall category structure. The planned ANOVA was conducted to evaluate the effect of group and feedback timing on slope scores. See Table 1 for full ANOVA results. Levene’s test was not significant (*p* = 0.15). The main effect of group approached but did not achieve significance (*p* = 0.056). The main effect of feedback timing was significant. Slope scores in the delayed condition indicated better learning (*M* = 7.40, *SD* = 4.14) compared to the immediate condition (*M* = 5.28, *SD* = 5.18). Differences across groups were revealed in the significant interaction between group and timing. Pairwise comparisons with a Holm correction revealed that older adults had slope scores closer to 10 in the delayed (*M* = 7.43, *SD* = 4.35) compared to the immediate condition (*M* = 3.19, *SD* = 5.54, *p* = 0.02). There was no difference in slope scores across timing conditions for younger adults (Delayed: *M* = 7.37, *SD* = 4.02, Immediate: *M* = 7.59, *SD* = 3.65, *p* = 0.8). These findings suggest that while older and younger adults performed comparably on the delayed feedback condition, older adults showed decreased learning under the immediate feedback condition (see Figure 5).

**Table 1:** 2 × 2 ANOVA results, with slope score as the dependent variable.

Predictor	*df_Num_*	*df_Den_*	*SS_Num_*	*SS_Den_*	*F*	*p*	η^2^_g_
(Intercept)	1	36	3099.77	820.34	136.03	<0.001	0.68
Age group	1	36	89.00	820.34	3.91	0.056	0.06
Feedback timing	1	36	76.17	624.53	4.39	0.043*	0.05
Age group × Feedback timing	1	36	94.33	624.53	5.44	0.025*	0.06

df_Num_ indicates degrees of freedom numerator. df_Den_ indicates degrees of freedom denominator. SS_Num_ indicates sum of squares numerator. SS_Den_ indicates sum of squares denominator. η^2^_g_ indicates generalized eta-squared.

* indicates significant effect.

### ERP data

#### FRN

Levene’s test was significant indicating unequal variances in FRN magnitude across the younger and older adults (*p* < 0.001). Thus, we were unable to conduct the planned mixed ANOVA with group as a between-subjects variable for the ANOVA with FRN factor score as the dependent variable. Instead, we conducted two separate ANOVAs for older and younger adults.

**Figure 6:**
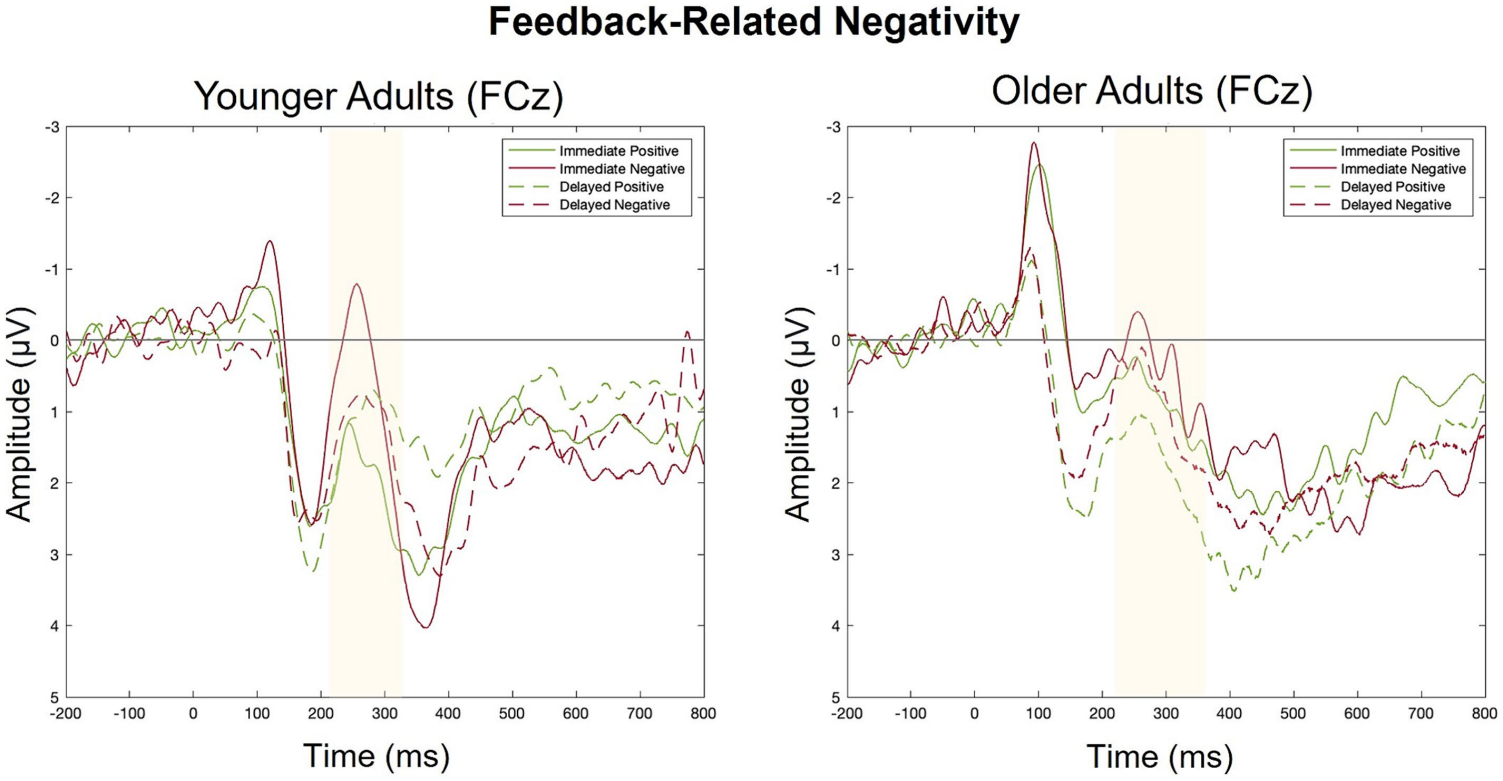
Grand averaged ERPs elicited by positive and negative feedback in Response to immediate and delayed feedback at FCz.

**Figure 7:**
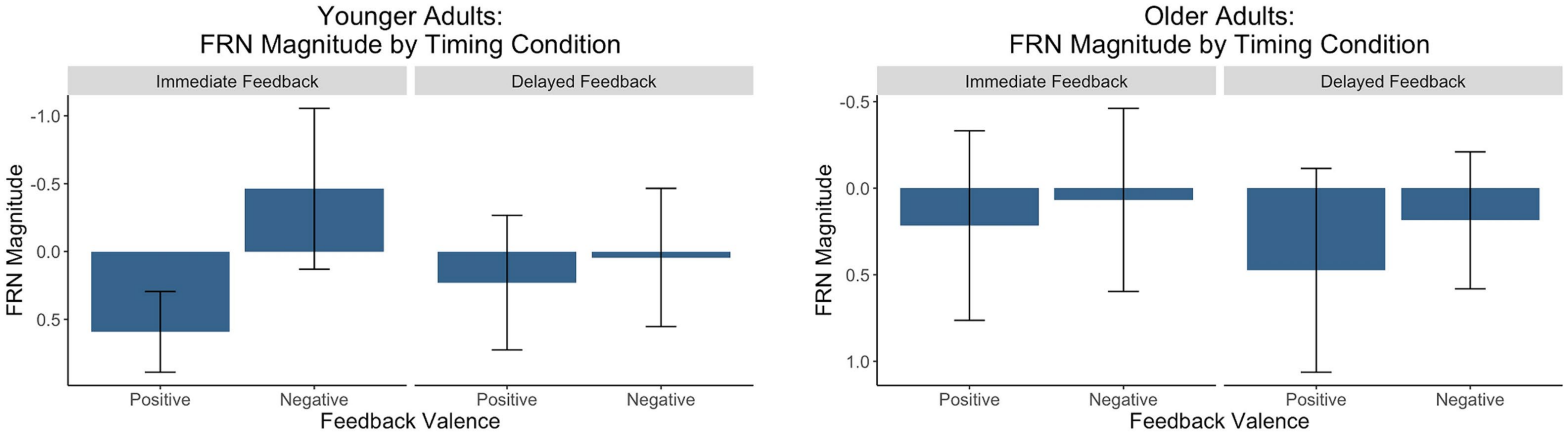
Factors scores reflecting the relative magnitude of the FRN. The *y*-axis is reversed because the FRN is a negative-going waveform.

For each group, we conducted a 2 (feedback timing: immediate vs. delayed) by 2 (feedback valence: positive vs. negative) ANOVA with FRN amplitude as the dependent variable. Figure 6 contains a grand-average waveform of the FRN by group. Figure 7 presents the factor scores which reflect the relative magnitude of the FRN. See Tables 2, 3 for the full ANOVA results.

**Table 2:** Young adult 2 × 2 ANOVA results, with FRN amplitude as the dependent variable.

Predictor	*df_Num_*	*df_Den_*	*SS_Num_*	*SS_Den_*	*F*	*p*	η^2^_g_
(Intercept)	1	16	0.69	20.42	0.54	0.47	0.01
Feedback timing	1	16	0.09	17.71	0.08	0.78	<0.001
Feedback valence	1	16	6.54	12.61	8.29	0.001*	0.10
Feedback timing × Feedback valence	1	16	3.21	6.42	8.01	0.001*	0.05

df_Num_ indicates degrees of freedom numerator. df_Den_ indicates degrees of freedom denominator. SS_Num_ indicates sum of squares numerator. SS_Den_ indicates sum of squares denominator. η^2^_g_ indicates generalized eta-squared.

* indicates significant effect.

**Table 3:** Older adult 2 × 2 ANOVA results, with FRN amplitude as the dependent variable.

Predictor	*df_Num_*	*df_Den_*	*SS_Num_*	*SS_Den_*	*F*	*p*	η^2^_g_
(Intercept)	1	16	3.78	43.98	1.38	0.26	0.05
Feedback timing	1	16	0.60	14.53	0.67	0.43	0.01
Feedback valence	1	16	0.81	4.17	3.12	0.10	0.01
Feedback timing × Feedback valence	1	16	0.08	2.81	0.47	0.50	<0.001

df_Num_ indicates degrees of freedom numerator. df_Den_ indicates degrees of freedom denominator. SS_Num_ indicates sum of squares numerator. SS_Den_ indicates sum of squares denominator. η^2^_g_ indicates generalized eta-squared. No significant effects observed.

#### FRN: younger adults

There was a main effect of feedback valence. The negative deflection of the FRN was larger for negative (*M* = −0.21, *SD* = 1.09) compared to positive (*M* = 0.41, *SD* = 0.80) feedback, as expected. The main effect of feedback timing was not significant. However, there was a significant interaction between timing and valence. Pairwise comparisons with a Holm correction revealed a significant difference between positive and negative feedback in the immediate (*p* = 0.006) but not the delayed (*p* = 0.3) timing condition. Thus, delaying feedback disrupted the typical signature of the FRN in which feedback is larger for negative relative to positive feedback.

#### FRN: older adults

There were no significant main effects. While numerically the FRN magnitude was larger for negative (*M* = 0.13, *SD* = 0.90) relative to positive (*M* = 0.35, *SD* = 1.10) feedback, this trend did not reach statistical significance indicating that older adults did not show the expected pattern of the FRN in either timing conditions.

#### *Post hoc* analyses at FCz

Upon visual inspection, it was observed that the older adult group appeared to show a larger N100 at FCz with immediate relative to delayed feedback. The N100 is evoked by visual stimuli and found to be larger to attended relative to unattended stimuli (Luck et al., 1994). To evaluate whether there was a significant difference in this negative deflection across conditions, we conducted a 2 (feedback timing: immediate vs. delayed) by 2 (feedback valence: positive vs. negative) ANOVA on the factor closest aligned with the negativity in older adults. There were no significant main effects or interactions.

#### N170

**Figure 8:**
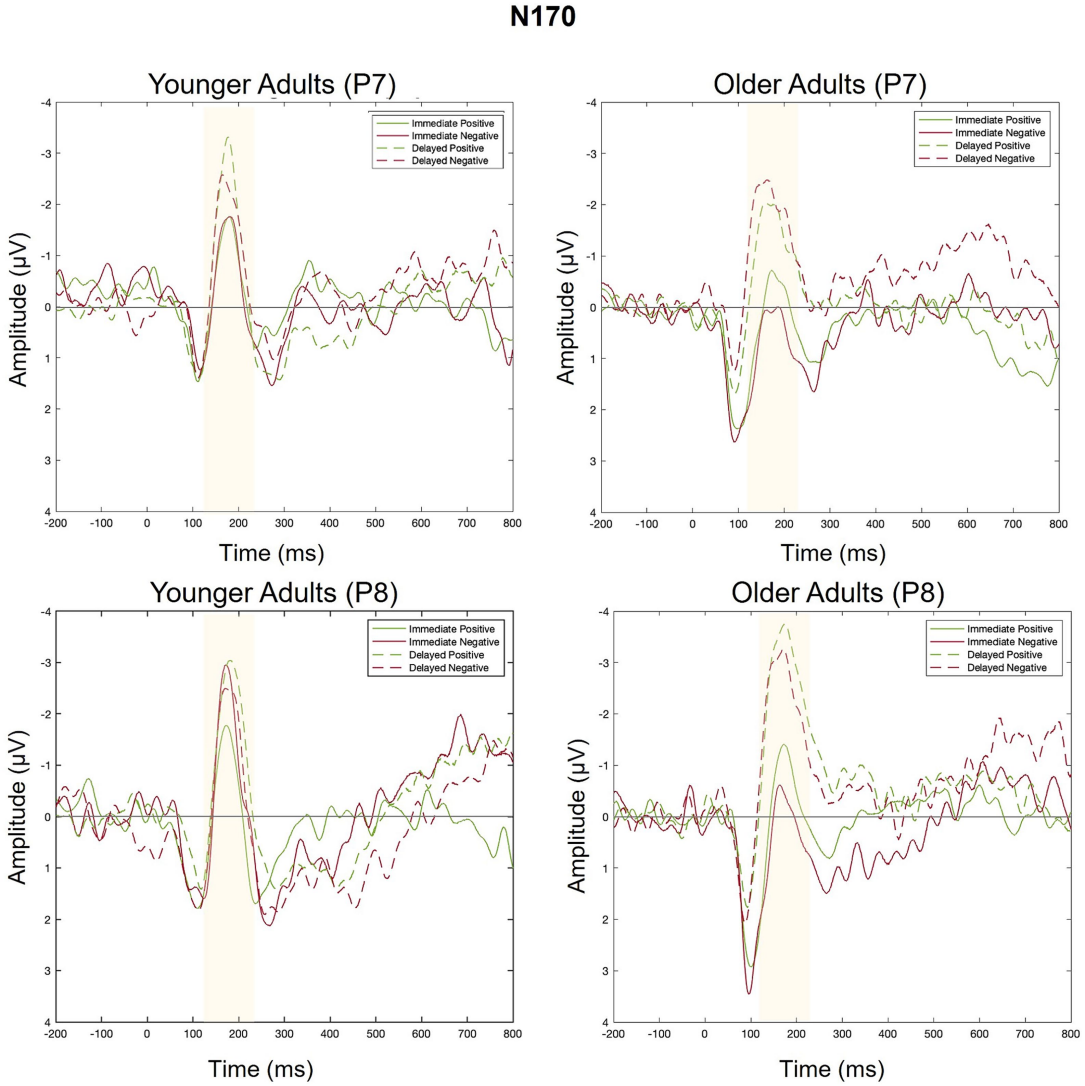
Grand average ERPs elicited by positive and negative feedback in Response to immediate and delayed feedback at electrodes P7 (left side of scalp) and P8 (right side of scalp).

**Figure 9:**
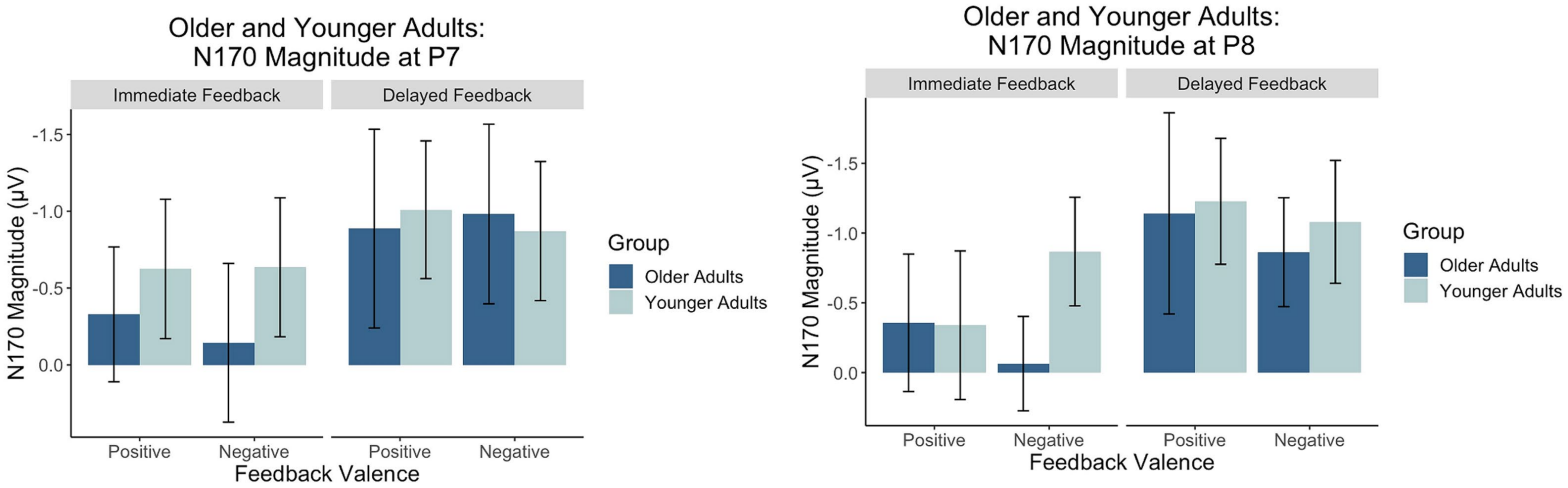
Factors scores reflecting the relative magnitude of the N170. Because the N170 is a negative-going waveform, the *y*-axis is reversed.

Levene’s test was not significant for P7 or P8 (*p* > 0.05), suggesting equal variance across groups. Figure 8 contains a grand-average waveform of the N170 by group and electrode. Figure 9 presents the N170 factor scores for each electrode and across conditions. See Tables 4 and 5 for full ANOVA results.

**Table 4:** 2 × 2 × 2 ANOVA results, with N170 amplitude at electrode P7 as the dependent variable.

Predictor	*df_Num_*	*df_Den_*	*SS_Num_*	*SS_Den_*	*F*	*p*	η^2^_g_
(Intercept)	1	32	63.87	69.83	29.27	<0.001	0.34
Age group	1	32	1.36	69.83	0.62	0.44	0.01
Feedback timing	1	32	8.66	40.45	6.85	0.01*	0.07
Feedback valence	1	32	0.10	6.70	0.49	0.49	0.00
Age group × Feedback timing	1	32	1.28	40.45	1.01	0.32	0.01
Age group × Feedback valence	1	32	0.00	6.70	0.01	0.91	0.00
Feedback timing × Feedback valence	1	32	0.04	6.20	0.18	0.67	0.00
Age group × Feedback timing × Feedback valence	1	32	0.39	6.20	2.03	0.16	0.00

df_Num_ indicates degrees of freedom numerator. df_Den_ indicates degrees of freedom denominator. SS_Num_ indicates sum of squares numerator. SS_Den_ indicates sum of squares denominator. η^2^_g_ indicates generalized eta-squared.

* indicates significant effect.

**Table 5:** 2 × 2 × 2 ANOVA results, with N170 amplitude at electrode P8 as the dependent variable.

Predictor	*df_Num_*	*df_Den_*	*SS_Num_*	*SS_Den_*	*F*	*p*	η^2^_g_
(Intercept)	1	32	74.98	65.53	36.62	<0.001	0.40
Age group	1	32	2.52	65.53	1.23	0.28	0.02
Feedback timing	1	32	15.32	25.87	18.95	<0.001*	0.12
Feedback valence	1	32	0.08	13.56	0.18	0.68	0.00
Age group × Feedback timing	1	32	0.49	25.87	0.61	0.44	0.00
Age group × Feedback valence	1	32	1.92	13.56	4.54	0.04*	0.02
Feedback timing × Feedback valence	1	32	0.93	7.78	3.81	0.06	0.01
Age group × Feedback timing × Feedback valence	1	32	1.01	7.78	4.14	0.05*	0.01

df_Num_ indicates degrees of freedom numerator. df_Den_ indicates degrees of freedom denominator. SS_Num_ indicates sum of squares numerator. SS_Den_ indicates sum of squares denominator. η^2^_g_ indicates generalized eta-squared.

* indicates significant effect.

#### P7

There was a main effect of feedback timing. The negative deflection of the N170 was larger for delayed (*M* = −0.94, *SD* = 1.03) compared to immediate (*M* = −0.43, *SD* = 0.91) feedback. There were no other significant main or interaction effects measured at P7.

#### P8

There was a main effect of timing. The negative deflection of the N170 was larger for delayed (*M* = −1.07, *SD* = 0.99) compared to immediate (*M* = −0.41, *SD* = 0.89) feedback. There were no other significant main effects. Differences across groups were revealed within interaction effects.

There was an interaction between group and valence. Pairwise comparisons revealed that the N170 was larger for younger compared to older adults when feedback was negative (Younger adults: *M* = −0.974, *SD* = 0.803; Older adults: *M* = −0.464, *SD* = 0.809, *p* = 0.02) but not when feedback was positive (Younger Adults: *M* = −0.78, *SD* = 1.05; Older Adults: *M* = −0.75, *SD* = 1.25, *p* = 0.09). There was also a significant three-way interaction. For older adults, N170 magnitude was larger for delayed relative to immediate feedback regardless of feedback valence. For younger adults, N170 magnitude was only larger for delayed relative to immediate feedback when feedback was positive.

#### *Post-hoc* analyses: P100 at P7 and P8

Upon visual inspection, it was observed that the older adult group appeared to show a larger P100 at P7 and P8 in response to immediate relative to delayed feedback. The P100 is thought to reflect top-down regulation of sensory information processed by the visual cortex (Luck and Kappenman, 2011). Within this interpretation, the P100 amplitude is expected to be larger for attended relative to unattended stimuli. To evaluate the potential for differences in attentional allocation across conditions we conducted a 2 (group: younger adults vs. younger adults) by 2 (feedback timing: immediate vs. delayed) by 2 (feedback valence: positive vs. negative) ANOVA on the factor closest aligned with the P100. Separate ANOVAs were conducted for P7 and P8. We found a main effect of feedback timing at P7 (*F*(1, 32) = 9.01, *p* = 0.005, η^2^ = 0.09) and P8 (*F*(1, 32) = 4.20 (*p* = 0.049, η^2^ = 0.05)). The positive deflection of the P100 was larger for immediate (P7: *M* = 1.0, *SD* = 1.0; P8: *M* = 1.1, *SD* = 1.06) relative to delayed (P7: *M* = 0.42, *SD* = 0.91; P8: *M* = 0.63, *SD* = 0.89) feedback. At P7, we also found a group by timing interaction (*F*(1, 32) = 4.5, *p* = 0.04, η^2^ = 0.04). The difference between immediate and delayed feedback was significant for older (*p* < 0.001) but not younger (*p* = 0.44) adults.

## Discussion

The current study aimed to evaluate how altering the timing of feedback influenced feedback processing and learning for younger and older adults. Behavioral data revealed that at the group-level participants learned the category structure as evidenced by an increase in %BResp when an exemplar shared more features with prototype B. A small subset of participants (~25%) did not show evidence of a linearly increasing relationship between %BResp and number of features shared with prototype B. Slope data allowed for the reduction of %BResp to a single score to evaluate response as a function of distance from the prototype. Slope score analysis revealed that older and younger adults had comparable learning in the delayed feedback condition but not the immediate feedback condition. Older adults performed worse in the immediate relative to delayed condition. Delaying feedback may warrant further exploration as a potential means to improve learning in older adults and potentially as a means to equalize performance across younger and older learners.

Electrophysiological data may further clarify behavioral findings. Younger adults showed the expected pattern in the FRN data. There was a larger FRN to negative compared to positive feedback in the immediate but not the delayed condition. These findings are consistent with fast-acting, dopamine-driven processing of immediate feedback that shifts to other circuits when feedback is delayed. Importantly, older adults showed a reduced FRN effect when learning from immediate feedback, suggesting a disruption of feedback processing in the striatum regardless of feedback timing. Age-related limits on older adults’ ability to effectively recruit the striatum to process immediate feedback may explain why younger adults outperformed older adults when feedback was immediate and dependent upon fast-acting subcortical reward processing (Nieuwenhuis et al., 2002; Eppinger et al., 2008).

The N170 amplitude was larger for both groups in the delayed relative to the immediate feedback condition. Again, this supports the notion that delaying feedback alters the neural processing of feedback and that this is captured by the N170. One potential neural generator is the MTL (Arbel et al., 2017; Kim and Arbel, 2019; Höltje and Mecklinger, 2020) which supports the integration of temporally discontiguous information. Specifically, research finds that the hippocampus contains “time cells” that encode key events that are separated by a temporal gap (MacDonald et al., 2011, 2013; Kitamura et al., 2015). In the context of the current task, the response (e.g., This animal lives in a cave) and feedback (e.g., incorrect) are temporally discontiguous events in the delayed condition (i.e., separated by a 6,000 ms time gap). What is known about the MTL and the demands of this task, make the MTL a potential candidate for the current task. However, further research is necessary to investigate the neural generator in this context and potential alternative sources (e.g., the fusiform gyrus).

Older adults learned better in the delayed relative to immediate condition suggesting that delaying feedback may be a fruitful way to support feedback-based learning in older adults. The locus of the gain in learning for older adults remains to be elucidated. One potential explanation is that delayed feedback allowed older adults to rely on the MTL to update cue-response contingencies when separated by a delay. The MTL may be better suited to support Prototype A/B learning in the setting of age-related neural changes compared to the striatum. While previous research has suggested a steeper age-related decline in the functioning of the MTL relative to the striatum in older adults, there is also a body of work which characterizes age-related dysfunction in reward processing within the striatal system with aging (Mell et al., 2005; Bäckman et al., 2006; Eppinger et al., 2008, 2013; Braver, 2012; Chowdhury et al., 2013; Samanez-Larkin et al., 2014). Changes in reward processing may also disrupt learning within the current task. A series of studies (Holroyd and Coles, 2002; Nieuwenhuis et al., 2002; Eppinger et al., 2008) have evaluated age-related reduction in feedback processing using the FRN. Eppinger et al. (2008) found that even when controlling for performance differences, older adults showed an atypical pattern in the processing of negative and positive feedback. The current findings are consistent with an age-related decline in dopaminergic processing reflected in symmetric processing of negative and positive feedback in older adults. Considering the current results within this context, age-related changes in the dopaminergic reward system may be more detrimental to performance on a Prototype A/B learning task than age-related changes in the MTL.

Alternatively, it could be that the delaying of feedback altered attentional recruitment in a manner that was advantageous for older adults. Post-hoc analyses revealed an interaction in which at P7 older, but not younger adults, showed a larger P100 for immediate relative to delayed feedback. Thus, immediate but not delayed feedback was associated with increased attentional allocation. One potential interpretation of this finding is that when older adults are unable to effectively rely on striatal mechanisms to process immediate feedback, more attention is allocated to the feedback signal.

Differences in demands on cognitive processing speed across the immediate and delayed feedback tasks may also contribute to age-related differences. Aging has long been associated with a gradual reduction in cognitive processing speed (Cerella and Hale, 1994). Delaying feedback may give adults sufficient time to process incoming stimuli and the resultant feedback despite reduced rates of processing. Potential explanations as to why older adults seemingly benefitted from delays in feedback timing are not mutually exclusive and warrant further evaluation to understand neural underpinnings.

The findings of the current study do not align with Lighthall et al. (2018) in which age-related decline was more evident in the hippocampal relative to striatal regions. Differences across studies may come from variations in the timing of immediate feedback. Immediate feedback was presented at 500 ms in the current study and at 1000 ms in Lighthall et al. (2018). Worthy et al. (2013) suggests that timing differences as small as 500 ms can affect learning by influencing the intracellular chemical concentrations at the time of the dopaminergic reward signal. In support of this hypothesis, Worthy et al. (2013) found that during an information integration category learning task accuracy was highest when feedback was presented at 500 ms compared to 0 ms or 1,000 ms. These findings suggest that differences in timing on the scale of milliseconds may influence learning driven by the striatum and could potentially explain differences in findings across otherwise comparable studies. Of course, other methodological differences across the current study and Lighthall et al. (2018) may also drive differences across studies; thus, future research should evaluate the reproducibility of the current findings. One such difference is the task type. We chose a prototype learning task given its relevance across the lifespan and that the effect of feedback timing has yet to evaluated. However, in a prototype A/B learning task, feedback may not be informative on a trial-by-trial basis and thus, affect the electrophysiological response to feedback.

If a consistent advantage for learning from delayed feedback is identified in older adults, or if individual differences arise, manipulating feedback timing may serve to optimize learning in this population. Techniques aimed at enhancing learning may be particularly useful for older adults with acquired neurologic impairments. Adults with acquired neurologic injury often receive rehabilitation services in which they re-learn skills and learn new compensatory strategies. Rehabilitation specialists must administer treatments in ways that target the impaired system but also successfully engage functioning systems of learning to induce neuroplastic change. As rehabilitation fields continue to move toward theory-driven treatments that delineate which treatment ingredients engage proposed mechanisms of action and how identified ingredients should be administered, feedback timing should not be overlooked.

### Limitations

In the current task, similar to probabilistic tasks, feedback was not intended to be useful on a trial-by-trial basis. Individuals needed to use feedback over the course of learning to slowly build a conceptual representation of the category structure. While this type of learning is similar to how humans acquire knowledge of complex category structures, the trial-by-trail utility of feedback may moderate FRN amplitude (Arbel et al., 2013, 2014). Future research evaluating the effect of manipulating feedback timing on feedback processing in declarative learning tasks may provide more insight into how feedback can be leveraged in contexts where adults make responses and receive useful corrective feedback on the accuracy of that response.

## Conclusion

This work suggests that in a prototype A/B category learning task, the timing of feedback (immediate vs. delayed) may have distinct consequences for learning in younger and older adults. Differences in learning across groups and timing conditions were associated with differences in electrophysiological response to feedback. Notably, older adults learned better from delayed relative to immediate feedback, potentially due to age-related changes in the neural mechanisms responsible for processing feedback. Future research is needed to determine the probable locus of the learning advantage with delayed feedback in older adults and can work toward understanding how feedback timing may be manipulated to promote learning across the lifespan.

## Data availability statement

The raw data supporting the conclusions of this article will be made available by the authors, without undue reservation.

## Ethics statement

The studies involving humans were approved by Institutional Review Boards of Mass General Brigham. The studies were conducted in accordance with the local legislation and institutional requirements. The participants provided their written informed consent to participate in this study.

## Author contributions

KN: Formal analysis, Investigation, Visualization, Writing – original draft, Writing – review & editing. RC: Conceptualization, Investigation, Methodology, Writing – review & editing. VT-B: Investigation, Writing – review & editing. YA: Conceptualization, Formal analysis, Funding acquisition, Methodology, Resources, Software, Supervision, Writing – review & editing. SV-R: Conceptualization, Formal analysis, Funding acquisition, Methodology, Resources, Supervision, Writing – review & editing.

## Footnotes

^1^The FRN has also been conceptualized as a “reward positivity” reflecting a reduction in the N200 magnitude following positive feedback due to (1) inhibition of processes eliciting the N200 or (2) a fronto-central positive deflection that overlaps in time with the N200 (Holroyd et al., 2008).
